# Wide sensory filters underlie performance in memory-based discrimination and generalization

**DOI:** 10.1371/journal.pone.0214817

**Published:** 2019-04-18

**Authors:** Chi Chen, Dilja Krueger-Burg, Livia de Hoz

**Affiliations:** 1 Department of Neurogenetics, Max Planck Institute of Experimental Medicine, Göttingen, Germany; 2 International Max Planck Research School Neurosciences, Göttingen Graduate School for Neurosciences and Molecular Biosciences, Göttingen, Germany; 3 Department of Molecular Neurobiology, Max Planck Institute of Experimental Medicine, Göttingen, Germany; Newcastle University, UNITED KINGDOM

## Abstract

The way animals respond to a stimulus depends largely on an internal comparison between the current sensation and the memory of previous stimuli and outcomes. We know little about the accuracy with which the physical properties of the stimuli influence this type of memory-based discriminative decisions. Research has focused largely on discriminations between stimuli presented in quick succession, where animals can make relative inferences (same or different; higher or lower) from trial to trial. In the current study we used a memory-based task to explore how the stimulus’ physical properties, in this case tone frequency, affect auditory discrimination and generalization in mice. Mice performed *ad libitum* while living in groups in their home quarters. We found that the frequency distance between safe and conditioned sounds had a constraining effect on discrimination. As the safe-to-conditioned distance decreased across groups, performance deteriorated rapidly, even for frequency differences significantly larger than reported discrimination thresholds. Generalization width was influenced both by the physical distance and the previous experience of the mice, and was not accompanied by a decrease in sensory acuity. In conclusion, memory-based discriminations along a single stimulus dimension are inherently hard, reflecting a high overlap between the memory traces of the relevant stimuli. Memory-based discriminations rely therefore on wide sensory filters.

## Introduction

An animal’s response to external stimuli depends largely on the animal’s capacity to identify the current stimulus as the same or similar to previously encountered stimuli. Often, experimental design is such that stimuli are presented in relatively quick succession (e.g. [1–4]). In these conditions the memory of the physical characteristics of the stimulus presented in the previous trial might still be active and a relative-judgement can be made. For example, the study of just-noticeable-differences (JNDs) or difference limens, i.e. the minimum physical differences that still allow discrimination, has received a lot of attention across modalities and is based on experiments that rely on relative-judgement [5–15]. The general finding is that across species, animals can make fine discriminations. In the auditory domain, for example, humans and rodents can differentiate between frequencies that differ in small ΔFs of a few percent [11–13]. Similarly, in the visual domain, orientations differing in a fraction of a degree can be discriminated [7,14,15]. From a different perspective, the study of absolute judgement [16], which tests the capacity of a subject to order a given stimulus among a group of stimuli varying along a single dimension, also relies on experimental designs in which stimuli are presented successively. In nature, however, animals often have to decide how to respond to a stimulus that is presented in spatial and temporal isolation from others that resemble it. This type of memory-based judgement is reflected in some forms of stimulus categorization, which has been the subject of substantial research in several species, including humans, monkeys and pigeons [17,18], but not in simpler discriminations. Understanding how perception of current stimuli is affected by the memory of other stimuli can help us understand the sensory filters that are in place during memory-based judgements, and infer the interaction between the involved neuronal populations. In the auditory domain, little is known about the role played by differences in a given physical dimension, such as sound frequency, in memory-based discriminations. In humans, tone frequency judgements, in isolation, are generally difficult if the subject lacks absolute pitch but can be ameliorated with the use of reference frequencies [19].

To further our understanding of the role played by the stimulus’ physical properties on memory-based discrimination in mice, we used sounds that varied along a single dimension, frequency, in a memory-based task in the Audiobox (TSE, Germany), an automatic apparatus in which mice live in groups and perform *ad libitum* for the duration of the experiment. We used two measures of performance to infer perception: discrimination learning and generalization of this learning to new and similar stimuli. While our main focus was on the role of a sound frequency distance in sound perception, we also measured the role of sound valence and previous training, as well as the effect that training had on spontaneous frequency discrimination using the startle reflex.

## Materials and methods

All animal experiments were approved by the local Animal Care and Use Committee (LAVES, NiedersaÈchsisches Landesamt fuer Verbraucherschutz und Lebensmittelsicherheit, Oldenburg, Germany) in accordance with the German Animal Protection Law. Project license number 33.14-42502-04-10/0288.

### Animals

Female C57BL/6JOlaHsd (Janvier, France) mice were used for the majority of experiments. Neuroligin 2 knockout (Nlgn2 KO) mice [20,21] were used in one test. Nlgn2 knockout mice were generated on a 129/Sv background and were backcrossed onto a C57BL/6J background for at least six generations. Female wildtype (WT) and homozygous (KO) littermates were obtained from Nlgn2 heterozygous breeding pairs. All mice were 5–6 weeks old at the beginning of the experiment. Animals were housed in groups and in a temperature-controlled environment (21 ± 1°C) on a 12 h light/dark schedule (7am/7pm) with access to food and water *ad libitum*. During behavioral training in the Audiobox (TSE, Germany), water was only available in the corners of the Audiobox (see below). Each mouse was lightly anaesthetized with Avertin i.p. (0.1ml/10g) and a sterile transponder (PeddyMark, 12 mm × 2 mm or 8 mm × 1.4 mm ISO microchips, 0.1 gr in weight, United Kingdom) was implanted subcutaneously in the upper back. Histoacryl (B. Braun) was used to close the small hole left on the skin by the transponder injection. These mice were allowed a minimum of 1 d for recovery before experimentation.

### Apparatus: Audiobox

All behavior was run in the Audiobox, a device developed for auditory research and based on the Intellicage (NewBehavior, Switzerland). Mice were kept in groups of 6 to 10 animals. Each animal was individually identifiable through the use of the implanted transponder. The Audiobox served both as living quarters for the mice and as their testing arena.

The Audiobox was placed in a soundproof room which was temperature regulated and kept in a 12 h dark/light schedule. The apparatus consists of three parts, a home cage, a drinking ‘corner’, and a long corridor connecting the other two parts (S1A Fig). The home cage serves as the living quarter, where the mice have access to food *ad libitum*. Water is available in two bottles situated in the drinking ‘corner’, which is positioned inside a sound-attenuated box. Presence of the mouse in the ‘corner’, a ‘visit’, is detected by an antenna located at the entrance of the corner. The antenna reads the unique transponder carried by each mouse as this enters the corner. A heat sensor within the corner senses the continued presence of the mouse. Identification of the specific mouse that enters the ‘corner’ is used to select the correct acoustic stimulus. Once in the ‘corner’, specific behaviors (nose-poking and licking) can be detected through other sensors. Access to water is controlled by opening or closing the doors behind nose-poking ports. The amount of water that a mouse can drink in each visit is not limited. Air puff is delivered through an automated valve which is placed on the ceiling of the ‘corner’. A loudspeaker (22TAF/G, Seas Prestige) is positioned right above the ‘corner’, for the presentation of sound stimuli. All behavioral data is logged for each mouse individually. We monitored the number of corner visits, nose-pokes and lick durations of each animal on a daily basis. During experimentation, cages and apparatus were cleaned once a week by the experimenter.

Sounds were generated using Matlab (Mathworks) at a sampling rate of 48 kHz. Stimuli consisted of 30 ms pure tone pips, with 5 ms rise/fall linear slopes, repeated at a rate of 3 Hz. Tones with frequencies between 4 and 18 kHz were used and presented in the corner throughout the visit. Stimuli presented within a given visit had the same frequency and intensities that roved between 67 and 73 dB.

### Memory-based discrimination task

**Fig 1:**
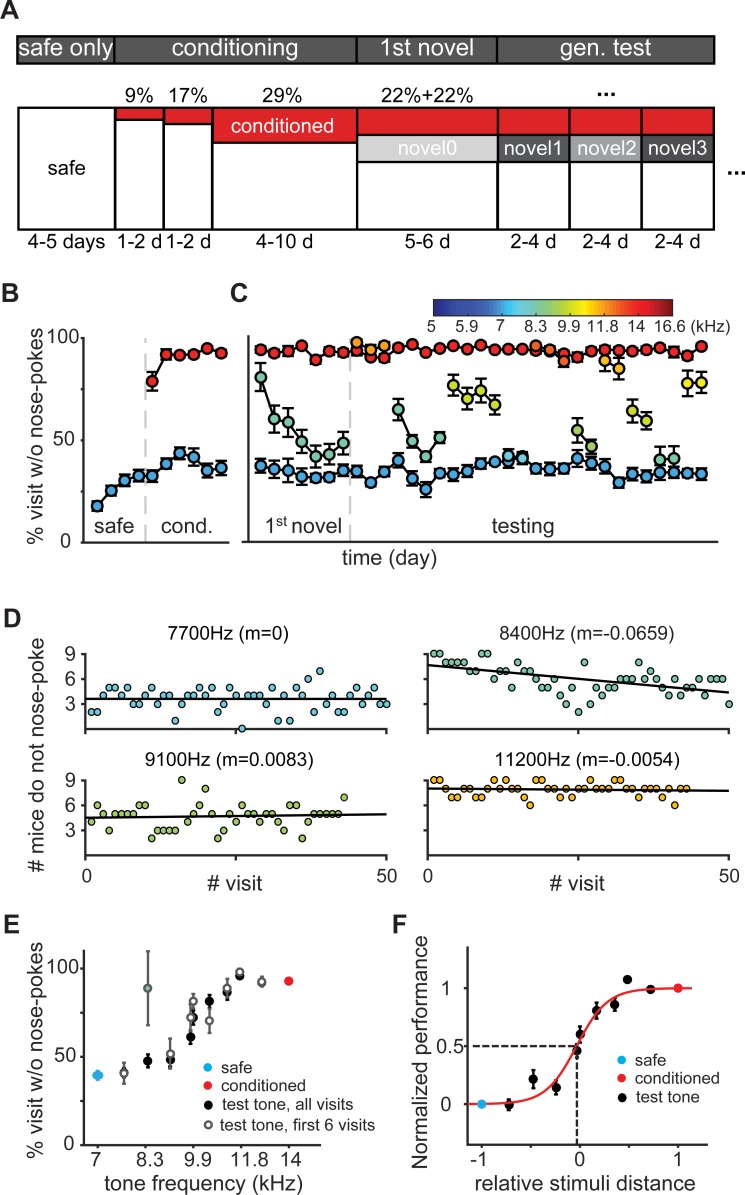
Discrimination training and generalization testing in the Audiobox. (**A**) Experimental design. The horizontal axis of the box represents time and the vertical axis represents the percentage of visits that were safe (white), conditioned (red), or novel (gray). (B) Performance during discrimination training. Mean daily performance expressed as the fraction of visits without nose-pokes for the safe (blue) and conditioned visits (red). (**C**) Performance during generalization test. Mean daily performance for the safe (blue), conditioned (red), novel visits (color-coded by novel tone frequency). (**D**) Single visit performance analysis as # mice (n = 9) that avoided nose-poking in each novel visit for novel visits with tones of 7700Hz (top-left), 8400 Hz (top-right, 1^st^ novel tone presented), 9100 Hz (bottom-left) or 11200 Hz (bottom-right). Linear regression shown as black line, and slope, on the title. (**E**) Generalization gradient, average performance as a function of tone frequency, for all visits (black) and the initial 6 visits of each visit type (white). Here and in subsequent figures, red and blue closed dots indicate responses to the conditioned and safe tone, respectively. (**F**) Normalized performance (black dots) and fitted psychometric curve (red). Evaluated psychometric threshold as the stimulus strength for which performance is at the midpoint (dash line).

The task resembles a go/no-go discrimination task with long inter-trial intervals. A ‘visit’ begins whenever an animal enters the corner and finishes only when the animal leaves the corner. During each visit, and for the duration of the visit, a single tone is played in pips of 30ms at a rate of presentation of 3 Hz. The tone stops playing when the animal leaves the corner. As soon as an animal leaves the corner, another visit might begin. Throughout the duration of the experiment, one frequency (i.e. 7000 Hz) was always ‘safe’, meaning that access to water during visits in which this tone was played was granted upon nose-poke. For the first 4 days only the safe tone was played in each visit (safe-only phase). The doors giving access to the water within the corner were open on the first day of training and closed thereafter. A nose-poke from the mouse, on either side of the corner, opened the door and allowed access to water. Then the conditioning phase started. A ‘conditioned’ tone, of a different frequency, was played in a small percentage of visits. Visits in which the conditioned tone was played are called ‘conditioned’ visits. A nose-poke during these visits immediately elicited an air puff and no access to water was granted in that the nose-poke doors remained closed throughout conditioned visits (S1B Fig). The probability of conditioned visits was 9.1% for 2 days, increased to 16.7% for 2 days, then stayed at 28.6% until mice showed steady discrimination performance (no significant change in group d’) for at least 3 consecutive days. The remaining daily visits continued to be safe (Fig 1A). Different groups of animals (8 to 10 mice per group) were trained with different pairs of safe and conditioned tones. The safe tone was either 5885 or 7000 Hz. And the conditioned tone was 0.25, 0.5, 075, or 1 octave higher than the safe tone (7000, 9800, 11770 Hz for 5885 Hz safe tone; 8320, 9800, 11770, or 14000 Hz for 7000 Hz safe tone). The experiments in which mice were trained with tone pairs of 7000–8320, 5885–7000 and 7000–14000 Hz were run over two replications.

Mice that failed to learn the task, defined by no differential responses to the safe and the conditioned tone for 3 consecutive days after one week of conditioning, were excluded from the analysis. In total, 19 out of 177 mice were excluded. All mice moved from phase to phase at the same time.

### Generalization gradients for tone frequency

Once the probability of conditioned visits reached 28.6% and animals showed stable discrimination performance, we tested generalization.

During generalization testing, we introduced novel tones in a small percentage of the visits. Novel tones differed from the safe and the conditioned tone in frequency. Nose poking during the presentation of the novel tone resulted in opening of the doors that gave access to water. Initially, only one novel tone was presented, with a tone frequency which was at a safe-to-conditioned distance of 25% from the safe and 75% from the conditioned frequency. The first novel tone was presented for 5–8 days until mice acquired stable performance in response to the novel tone. The remaining novel sounds were introduced in pairs, two per block, with pseudo-random order and tested for 4 days each (~50 visits). A mouse would be thus exposed to 55.6% of safe visits, 22.2% of conditioned visits and 22.2% of novel visits (11.1% for each of the two novel sounds) per day. In one of the replications in which mice were trained with the 7000–14000 Hz pair, novel tones were presented singly for 2–3 days each with a probability of appearance of 22.2% (Fig 1A).

### Analysis of performance in the Audiobox

Data were analyzed using in-house scripts developed in Matlab (Mathwork). Performance traces for different stimuli were calculated by averaging the fraction of visits without nose-pokes over a 24-hour window. Discrimination performance was quantified by the standard measures from signal detection theory, the discriminability (d’). It was calculated with the assumption that the decision variables for the safe and conditioned tone have a Gaussian distribution around their corresponding mean and comparable variance. The d’ value provides the standardized separation between the mean of the signal present and the signal absent distribution. It is calculated as:

$$d^{\prime }=Z(HR)-Z(FAR)$$

Where Z(p), p ϵ [0 1], is the inverse of the cumulative Gaussian distribution, HR is the hit rate, where a hit is the correct avoidance of a nose-poke in a conditioned visit, and FAR is the false alarm rate, where a false alarm is the avoidance of a nose-poke in a safe visit. Since d’ cannot be calculated when either the false alarms reach levels of 100% or 0%, in the few cases where this happened we used 95% and 5% respectively for these calculations. This manipulation reduced d’ slightly, and therefore our d’ estimates are conservative.

To evaluate the psychometric threshold and slope, we fit a sigmoid function to the normalized performance. Performance was normalized independently for each mouse. The mean percentage of visits without nose-pokes, overall testing days including the first novel sound test, was set to 100% for the conditioned visits and to 0% for the safe visits. The conditioned-to-safe frequency distance was also normalized such that the safe pure tone was set to -1 and the conditioned tone was set to 1. Stimuli were represented by their relative distance to the safe (-1) and conditioned (1) tones. Relative stimuli distance was calculated as:

$$\Delta S=\frac{\left(f-f_{s})+(f-f_{c}\right)}{f_{c}-f_{s}}$$

Where *f* is the frequency of the stimulus, *f*_*s*_ is the frequency of the safe tone and *f*_*c*_ is the frequency of the conditioned tone. In analysis for the retraining, since more than one conditioned tone was used, fitting was done to the performance in response to different frequency. Briefly, a constrained maximum likelihood method was used to fit a logistic function with 4 parameters: α (the 50% threshold), 1/β (the slope of the curve), γ (the lower asymptote), and λ (the higher asymptote).

$$\psi (x)=\gamma +(1-\gamma -\lambda )\frac{1}{1+\exp\left(-g(x)\right)}$$

$$g(x)=\frac{x-\alpha }{\beta }$$

Animals with a goodness of fit (R^2^) below 0.7 were excluded from statistical analysis of threshold and slope. This was the case for 15 out of 118 animals.

### Frequency discrimination acuity test

We used a modified pre-pulse inhibition (PPI) of the startle reflex protocol to measure frequency discrimination acuity as previously described [22,23]. Measurements were performed in a sound attenuated room. A schematic of the experiment setup is illustrated in S5A Fig. The sound was synthesized using Matlab (Mathworks), and played in a free-field 705 ultrasonic speaker (Ultrasonic Dynamic Speaker Vifa, Avisoft, Germany) through an interface (Octa capture, Roland, 704 USA) and an amplifier (Portable Ultrasonic Power Amplifier, Avisoft Germany). Simultaneously generated triggers were detected through an analog-to-digital converter data acquisition system (NI SCB-68, National Instruments, TX). Animal was positioned in a custom-made chamber adjusted to the size of the mouse (length 10 cm, inner diameter 4.2 cm, outer diameter 5.0 cm). The chamber rested upon a piezoelectric sensor (30 V, 717770, TRU COMPONENTS) for movement detection. The speaker was placed 15 cm away from the head of the animal.

The startle stimulus was a 40 ms broad-band noise at 105 dB SPL. A background tone (f1, 70dB SPL) was presented continuously between the end of startle stimulus and the start of the pre-pulse stimulus. The pre-pulse stimulus (f2, 70dB SPL) was 80 ms long and consisted of a 1 ms linear ramp from background tone, f1, to the pre-pulse tone, f2. In each session, 13 frequencies were used as pre-pulse stimuli, corresponding to frequency changes (Δf = log2 (f2/f1)) of -0.56, -0.25, -0.12, -0.07, -0.03, -0.01, 0, 0.01, 0.03, 0.07, 0.11, 0.21 and 0.40 octave, respectively.

At the start of each session, the mouse was placed in the chamber and allowed to habituate for 5 min. This was followed by another 5 min of acclimation to a constant background tone (f1). The acclimation period was followed by 10 startle-only trials, 130 pre-pulse trials, and lastly by 10 startle-only trials. In startle-only trials, startle stimulus appeared directly after the background tone. In pre-pulse trials, the startle sound was immediately preceded by one of the pre-pulse stimuli. Each pre-pulse stimulus was presented 10 times in pseudo-random order. All trials had lengths varying randomly between 10 and 20 s.

The amplitude of acoustic startle response (ASR) was measured as the maximal vertical force exerted by the animal on the sensor during the 500 ms window beginning at startle stimulus onset minus the average baseline activity in the 500 ms window before the pre-pulse stimulus. The level of startle-only ASRs was calculated by averaging ASRs after f1 in the startle only trials. To calculate PPI, the 7 strongest ASRs for each pre-pulse stimulus out of 10 were averaged. The level of inhibition for each pre-pulse frequency was calculated as follows:

$$PPI(\%)=100\times \frac{ASRp{ps}_{f1}-ASRpps}{ASRp{ps}_{f1}}$$

In which ASRpps_f1_ is the response when pre-pulse frequency is equal to the background frequency and ASRpps is the response after pre-pulse stimulus. Discrimination threshold was defined as a frequency shift that elicited 50% of the maximum inhibition, calculated from a parametric fit to a generalized logistic function:

$$PPI=-\frac{a}{2}+\frac{a}{1+exp(b+c\Delta f)}$$

The fitting was done separately for pre-pulse frequency higher or lower than the background frequency (S7C Fig). Curves with a fit coefficient (R^2^) below 0.6 were excluded from statistical analysis. 7 lower curves and 13 upper curves out of 40 were excluded.

### Statistical analysis

**Fig 2:**
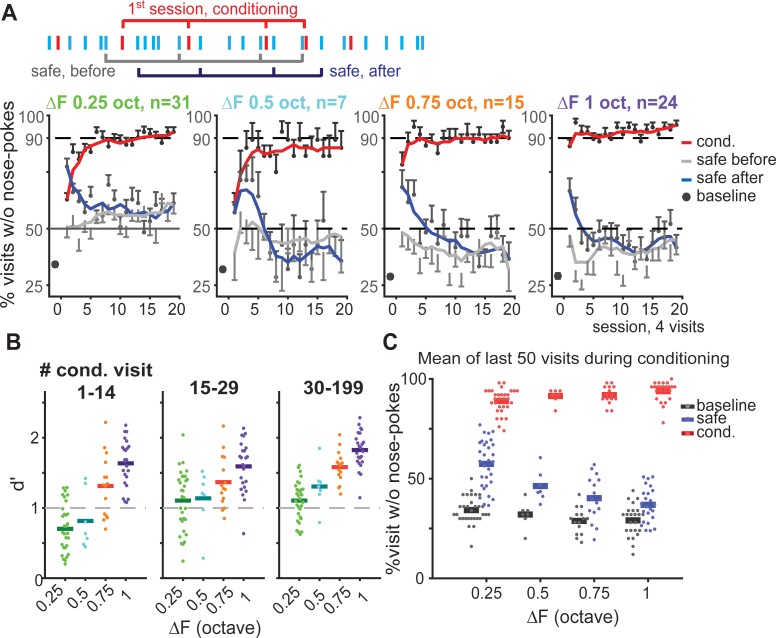
Discrimination performance deteriorated as the safe-to-conditioned ΔF decreased. (**A**) Top: Schema of learning curve analysis in sessions: average of blocks of 4 visits starting after the first conditioned visit (red) separately for safe visits (blue) that follow conditioned visits (blue brackets), safe visits that precede conditioned visits (gray brackets), and conditioned visits (black brackets). Bottom: performance analysis pooled across groups trained with same safe-to-conditioned ΔF, (ΔF 0.5 group, 1 replication only). Baseline performance (black dot) calculated as the mean response over the last 50 safe visits before the first conditioned visit. Mean performance in each session was plotted using a smoothed learning curve (5 moving average filter) for safe visits following conditioned visits (dark blue), safe visits preceding conditioned visits (gray), and conditioned visits (red). Non-smoothed data points are shown in the background as half error bars in different shades of gray. (**B**) d’ calculated as a function of the ΔF for conditioned visits number 1–14 (left), 15–29 (middle) and 30–199 (right) starting after the first conditioned visit. (**C**) Mean responses to the safe (blue) and conditioned (red) tones during the last 50 conditioned visits in the conditioning phase across groups trained with different ΔFs. Baseline (black) was calculated the same as (**A**). Here and in subsequent figures, dots in scatter plots represent result for an individual mouse and horizontal bars describe average across animals.

**Fig 3:**
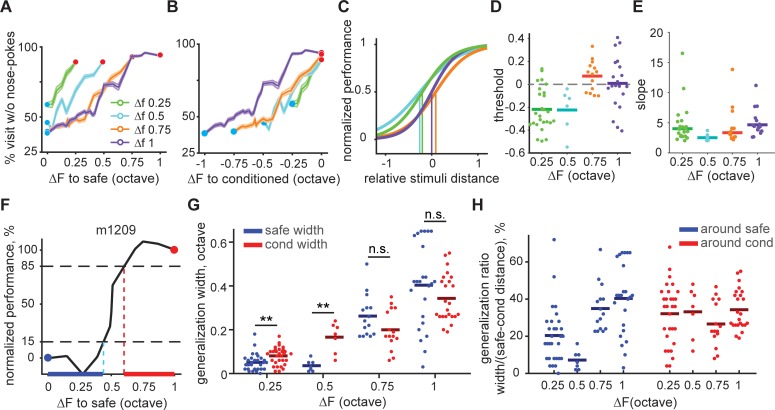
Asymmetrical generalization gradients in mice trained with small ΔFs. (**A**) Average performance as a function of frequency distance to the safe tone across groups trained with different ΔFs. (**B**) Average performance as a function of frequency distance to the conditioned tone across groups trained with different ΔFs. (**C**) Fitted psychometric curves and thresholds (vertical line). (**D-E**) Individual mouse threshold and slope of the psychometric curves in (**C**). (**A-E**) Color coding is based on the ΔF. (**F**) Example generalization width around the safe (blue line) and conditioned (red line) tone estimated for one mouse. (**G**) Generalization width for all mice. Here and in subsequent figures, *p < 0.05, **p < 0.01, ***p < 0.001, ****p < 0.0001; n.s., not significant (p > 0.05). (**H**) Relative generalization width as percentage of the safe-to-conditioned distance for all mice.

**Fig 4:**
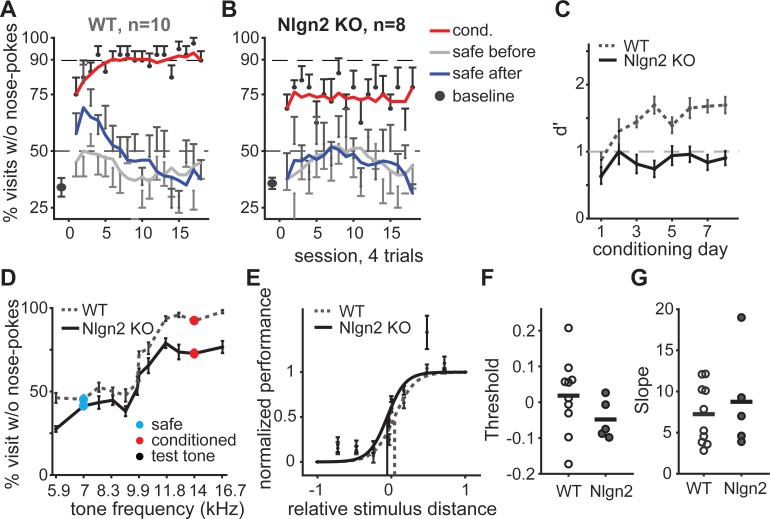
Neuroligin 2 knockout mice showed impaired discrimination but normal generalization gradients. (**A**) Baseline performance (black dot) and learning curves for Nlgn2 WT mice. (**B**) Same as (**A**) for Nlgn2 knockout mice. (**C-E**) Comparison between Nlgn2 WT (gray dash line) and knockout (black solid line) mice. (**C**) Mean d’ calculated as a function of conditioning day. (**D**) Generalization gradients based on average performance as a function of visit tone-frequency. (**E**) Fitted psychometric curves and thresholds (vertical line). (**F-G**) Threshold and slope of the psychometric curves, respectively. Mice did not pass the goodness of fit were excluded in the individual plot.

**Fig 5:**
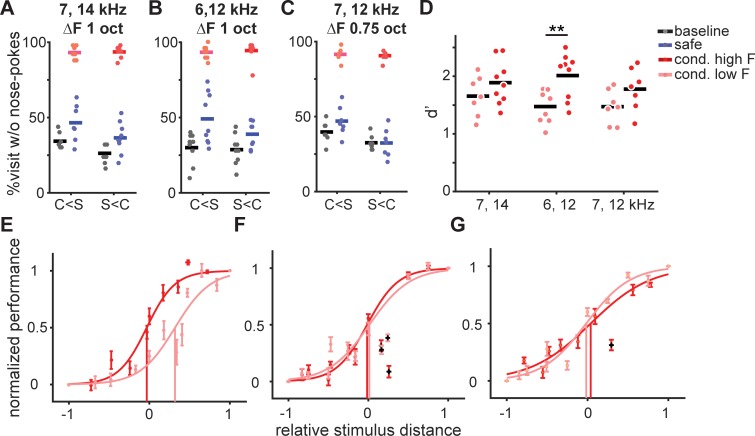
The direction of conditioning along the frequency axis affected learning, but not generalization. (**A-C**) Comparison between mice trained with different direction of conditioning (conditioned tone was higher or lower than safe) along the frequency axis. Baseline (black) and average responses to the safe (blue) and conditioned (pink/red) tones during the last 50 conditioned visits in the conditioning phase. All mice in a plot were trained with the same pair of stimuli, (A) 7000 Hz and 14000 Hz tone, (**B**) 5885 Hz and 11770 Hz tone, and (**C**) 7000 Hz and 11770 Hz tone. (**D**) Mean d’ during the last 50 conditioned visits in the conditioning phase. Stars: p = 0.004 2-sample t-test (**E-G**) Fitted psychometric curves and thresholds (vertical line) for mice described in (**A-C**), respectively. Black dots indicate outliers in the fitting (value above or below 1.5 times the standard deviation).

**Fig 6:**
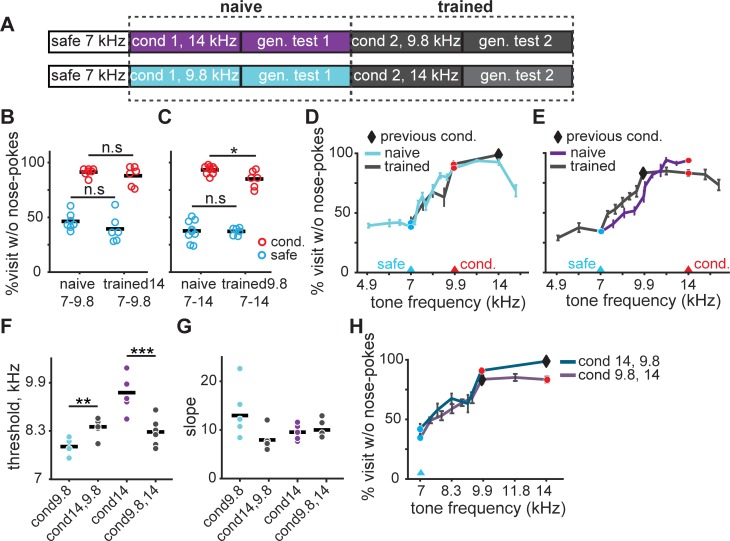
Previous task experience did not facilitate discrimination but shifted generalization gradients. (**A**) Experimental design: two groups of mice were initially conditioned with 14000 Hz or 9800 Hz tone, and then retrained to 9800 Hz or 14000 Hz tone, respectively. The safe tone remained at 7000 Hz throughout. Generalization gradients were measured following each conditioning. (**B**) Comparison of discrimination performance between naïve (1^st^ conditioning) and trained (2^nd^ conditioning) mice conditioned to 9800 Hz tone. Average responses to the safe (edge color blue) and conditioned (edge color red) tones during the last 50 conditioned visits in the conditioning phase. (**C**) Same as (**B**) for naïve and trained mice when conditioned to 14000 Hz tone. (**D**) Generalization gradients for naïve and trained mice following conditioning to a 9800 Hz tone. (**E**) Same as (**D**) for mice following conditioning to a 14000 Hz tone. (**F-G**) Threshold and slope of the psychometric curve for all mice in each testing. (**H**) Comparison of generalization gradients between mice conditioned to 14000 Hz then 9800 Hz and those conditioned to 9800 Hz then 14000 Hz. (**D, E, H**) The safe (blue circle), conditioned (red circle), as well as previously conditioned tone (black diamond) was marked respectively.

**Fig 7:**
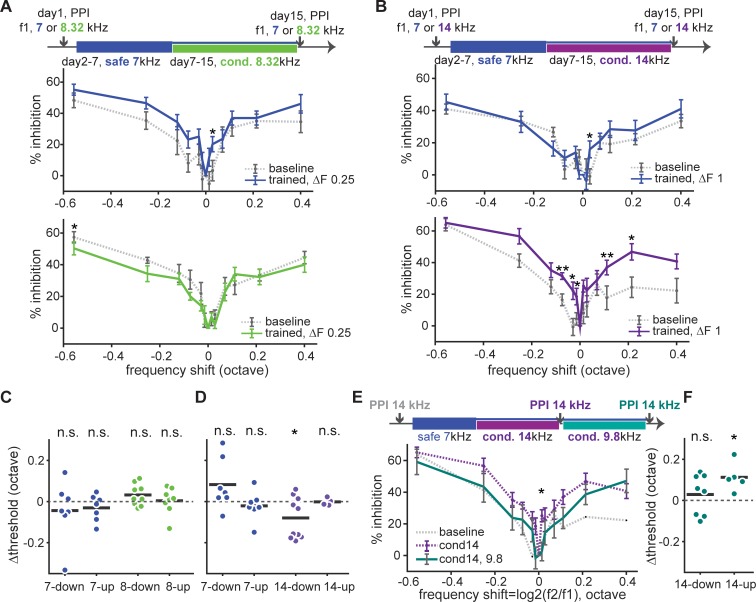
Audiobox learning led to ΔF-specific increase in discrimination acuity around the conditioned tone. (**A-top**) Experimental design: mice were tested for PPI-based discrimination acuity both before and after the Audiobox training. Two groups of mice were tested separately using either the safe or the conditioned tone as the background sound in both PPI tests. (**A-middle**) Baseline PPI (gray dash line) and PPI following Audiobox training (blue solid line) as a function of frequency shift from background (f1) where f1 was the same frequency as the safe tone (7000 Hz). (**A-bottom**) Baseline PPI (gray dash line) and PPI following Audiobox training (green solid line) as a function of frequency shift from f1 where f1 was the frequency as the conditioned tone (8320 Hz). (**B**) Same as (**A**) for mice conditioned to 14000 Hz. (**C**) Change in discrimination threshold around the safe (7000 Hz) and conditioned (8320 Hz) tone following Audiobox training. Calculation was done separately for f2 lower (down) and higher (up) in frequency than the background tone. (**D**) Same as (**C**) for mice trained with 7000 Hz and 14000 Hz tone. (**E-top**) Experimental design: mice went through two conditioning and PPI was tested before and after each conditioning using 14000 Hz as background tone throughout. (**E-bottom left**) Baseline PPI (gray dash line) and PPI following the first (purple dash line) and second (cyan solid line) conditioning as a function of frequency shift when f1 was the same in frequency as the first conditioned tone (14000 Hz). (**E-bottom right**) Change in Discrimination threshold around the first conditioned (14000 Hz) tone following the second conditioning.

Group comparisons were made using multiple way ANOVAs after testing for normality distribution using the Shapiro-Wilk test. All data were normally distributed. For analysis of data consisting of two groups we used either paired t-tests for within-subject repeated measurements or unpaired t-tests otherwise. For data consisting of more than two groups or multiple parameters we used, repeated-measures ANOVA. All multiple comparisons used critical values from a t distribution, adjusted by Bonferroni correction with an alpha level set to 0.05. Statistical significance was considered if *p* < 0.05. Means are expressed ± SEM. Data analysis was performed in MATLAB (Mathworks, USA). Minimal data sets are available in S1 File for all plots in Figs 1 to 7, and in S2 File for all plots in S1 to S7 Figs.

## Results

C57BL/6JOlaHsd mice were trained to perform auditory tasks in a behavioral apparatus, the Audiobox (TSE; S1A Fig), in which mice live for the duration of the experiment (several weeks) while performing the task *ad libitum*. The apparatus consisted of two areas, a food area where food was present all the time, and a corner within a sound-attenuated box. Mice could obtain water only in the ‘corner’ of the Audiobox (S1A Fig), a small enclave with two water spouts. A visit to the corner is defined by the time the mouse spends in the corner between an entry and an exit. Typically, mice made about 120 visits per day (S1C Fig; [24]). On each visit to the corner, and for the duration of the visit, mice were presented with a sound stimulus consisting of a train of pure tone pips of the same frequency. Initially, we trained mice in a pure-tone frequency discrimination task that resembled a go/no-go discrimination paradigm. Importantly, most visits, over two thirds, are spaced by intervals of more than 1 minute (S1D Fig; [25]) and tone frequency judgement must, therefore, rely on memory. In those visits in which the ‘safe sound’ was presented (safe visits), mice had access to water through a nose-poke on either side of corner (S1B-top Fig), whereas when the ‘conditioning sound’ was played (conditioned visits), nose-poking on either side was followed by an aversive air puff and no access to water (S1B-bottom Fig).

### Mice associated acoustic stimuli with different behavioral outcomes and generalized the learnt association to novel stimuli

The frequency discrimination paradigm used in our experiments is outlined in Fig 1A and S1B Fig. In the first experiment, mice (n = 9) were trained with two frequencies 1 octave apart: 7000 Hz as the safe sound and 14000 Hz as the conditioned sound. Conditioning visits were introduced after a 4-day phase of only safe visits. We calculated the average percentage of visits without nose-pokes across mice in blocks of 24 hours separately for safe and conditioned visits as a main performance index (Fig 1B). On average, mice did not nose-poke in about 30% of the safe visits, which is the typical baseline performance of the Audiobox [24]. This high rate of false alarms results from ad libitum behavior in the absence of water deprivation and continuous availability of water 24 hours per day. Visits without nose-pokes were always short (< 10 seconds; S1E Fig left). Once conditioning began, mice learnt the task fast and discriminated successfully as revealed by a clear nose-poke avoidance during conditioned visits and a significant difference in the nose-poking behavior during safe and conditioned visits (6 days; 3-way ANOVA on tone frequency, animal and day, revealed a main effect of frequency *F*(1, 93) = 1110.41, *p* = 0; effect of animal *F*(8,93) = 2.78, *p* = 0.0084; effect of day *F*(5,93) = 5.1, *p* = 0.0004). Conditioned visits were typically short (S1E Fig right). In the rare conditioned visits in which the animals nose-poked, the latency to nose-poke correlates well with the length of the visit (see Fig 3 in [24]). Already in the first day of conditioning, avoidance responses to the conditioned tone reached 79%. This increased to 94% on the following day, while the avoidance responses to the safe tone remained approximately at 35% throughout (Fig 1B). The training continued until the probability of conditioned visits reached 29% and mice achieved a stable discrimination performance, defined by a lack of significant change in group d’ value for at least three consecutive days (*p* > 0.05).

To measure generalization, we introduced ‘novel’ tones in 22% of the total daily visits. During these visits a novel tone was presented (S1B-top Fig). Safe visits and conditioned visits constituted now 56% and 22% of the total visits respectively (Fig 1A) and the animals were required to continue to discriminate between the safe and conditioned tones. Novel visits were actually safe, and nose-poking on either side resulted in opening of the nose-poke door and access to water. These novel tones were typically of a frequency somewhere in-between the safe and conditioned sounds and were introduced in a pseudo-random order (see Methods for detail). We started with a novel tone of 8400 Hz in frequency, equivalent to 0.26 octave above the safe sound. Because the conditioned sound is different in both frequency and probability of appearance, mice often treat other rare sounds as conditioned [24]. For this reason, the first novel sound was relatively close in frequency to the safe sound and was presented during at least 6 consecutive days until the animals reached a stable response in its presence. Indeed, mice avoided nose-poking during novel visits on the first day (Fig 1C, 1^st^ novel). But this avoidance gradually decreased and stabilized around 50%. Fig 1C shows the number of mice that avoided nose-poking in each individual novel visits for different frequencies. It is clear that while most mice avoided nose-pokes on the first presentations of the first novel sound, avoidance decreased over successive visits (Fig 1D, 8400 Hz). For the remaining novel sounds, the level of avoidance tended to be either low or high (Fig 1C, testing). This was already visible from the first visit on which a novel sound was presented, and it remained at that level throughout (Fig 1D; 7700 Hz, 9100 Hz and 11200 Hz), suggesting that the decision whether to approach or avoid the water port was made based on the frequency of the sound. The introduction of novel sounds did not affect the level of performance during either safe or conditioned visits (Fig 1C, blue and red points respectively).

To better visualize the data, we constructed psychometric curves for individual mice by plotting average percentage of visits without nose-pokes against the tone frequency. The psychometric curve took the shape of a sigmoid-function. Individual animals showed similar level of generalization around trained frequencies and more variable performance in-between them (S2A Fig). Mice showed generally a high level of avoidance responses to novel tones with high frequency and low level avoidance to tones with low frequency (Fig 1E), and this effect was present already during the first 6 visits of each sound-frequency (open dots). A 2-ways ANOVA on tone frequency and animal, revealed a main effect of frequency (*F*(10, 80) = 66.73, *p* < 0.0001) and animal (*F*(8, 80) = 6.32, *p* < 0.0001). Therefore, learning generalized from the trained sounds to the other tones in a frequency-specific manner, independently of the fact that water was available during all novel visits and no air-puff was delivered. For frequencies below 7700 Hz or above 11200 Hz, mice responded as if those visits were equivalent to safe or conditioned visits, respectively (Fig 1E). A one-way ANOVA, found a strong main effect of tone frequency (*F*(88,10) = 41.41, *p* < 0.00001). Multiple comparisons revealed that responses to 7000, 7700, 8400, and 9100 Hz were not significantly different from each other, and responses to 10500, 11200, 11700, 12700, 14000 Hz did also not differ from each other.

We estimated the subjective generalization threshold by fitting a logistic function to the normalized psychometric performance and calculated the psychometric threshold, i.e. the relative stimulus distance for which performance is at the midpoint. The psychometric threshold was -0.01, a stimulus level almost equidistant to the safe and conditioned frequencies (Fig 1F). This indicates that generalization around the safe and conditioned stimuli was symmetrical.

To understand whether learning and generalization were reflected in other aspects of the behavior other than nose-poking avoidance, we used the duration of the visit as an index of performance. The average duration of visits in which mice did not nose-poke was relatively short, approximately 3 seconds, independently of whether visits were conditioned, safe, or novel (S2B Fig, empty circles). However, for visits in which mice made nose-pokes, the psychometric curve of visit length was again sigmoidal. Conditioned visits with nose-pokes were longer than visits without nose-pokes, reflecting the fact that mice were unsure before they made an error and nose-poked (S2B Fig, filled circles). The normalized psychometric curve was identical to the one shown before (S2C Fig; compare with Fig 1E).

### Discrimination task performance deteriorated as the safe-to-conditioned ΔF decreased

We next investigated how discrimination between positive and negative stimuli was influenced by the physical distance between trained stimuli. The frequency distance between the safe and the conditioned tone was calculated in octaves, defined as ΔF. We trained different cohorts of naïve mice (*n* = 77) to discriminate between safe and conditioned tones that were 0.25, 0.5, 0.75 or 1 octave away from each other (with 4, 1, 2 and 3 replications respectively). The safe tone was either 5885 Hz or 7000 Hz and the conditioned tone was above the safe tone in frequency. Cohorts have been grouped according to ΔF since there was no difference in the behavior for different safe tones. The animals already described above are included within the 1 octave group.

Animals in all groups learnt the task fast and successfully and achieved a significant difference in the nose-poking behavior between safe and conditioned visits in the conditioning phase. To compare the efficiency of learning, we calculated the average percentage of visits without nose-pokes in blocks of 4 visits starting after the first conditioned visit (Fig 2A). We did this separately for conditioned visits (red), safe visits that followed a conditioned visit (blue), and safe visits that preceded one (gray). The division of safe visits intended to test the effect of a conditioned visit on the safe ones. When comparing across groups, it became evident that learning to avoid nose-poking in conditioned visits was faster for larger safe-to-conditioned ΔFs and that the time required to meet the criterion of stable avoidance of the conditioned tone (90% of visits without nose-poke) increased as the ΔF decreased.

One interesting finding was that discrimination learning was not limited to conditioned visits. The behavior of the mice in the safe visits during the conditioning phase was affected as well. Following each conditioned visit, mice showed increased avoidance of nose-poking in the subsequent safe visit compared with the baseline performance before conditioning began (Fig 2A, blue line vs baseline). However, this increase was temporary and gradually diminished reaching a plateau after about 20 conditioned visits. In animals trained with the smallest ΔF, but not the other groups, the plateau remained above baseline throughout (Fig 2A, left, gray and blue line).

Thus, as expected, it took longer time for mice trained with smaller ΔFs to learn to discriminate safe from conditioned sounds. This was reflected in the d’ values (Fig 2B), a standard measurement of discriminability adopted from signal detection theory, reflecting the separation between the mean of the correct target responses and the false alarms. While animals trained to discriminate across larger ΔFs showed d’ values well above 1 already in the first 15 visits, animals trained with smaller ΔFs required progressively more visits or never achieved this level. This resulted from a combined effect of the initial low nose-poke avoidance during the conditioned visits and the increased nose-poke avoidance during safe visits with respect to other groups, as reflected in Fig 2B.

The effect that conditioning had on the safe visits was clear even after overtraining. We quantified animals’ steady responses during safe and conditioned visits using the average performance during the last 50 conditioned visits window (Fig 2C; ca. last 2 days of conditioning, after 80 to 300 conditioned visits). The baseline performance, which was calculated as the mean performance during the last 50 visits in the safe phase (gray dots), was not significantly different between groups (*p* > 0.05). For both safe and conditioned visits, we observe significant difference in nose-poking behavior between the groups (1-way ANOVA of performance on delta f: for the safe visits *F*(3,73) = 19.25, *p* < 0.0001 and for the conditioned visits *F*(3,73) = 4, *p* = 0.011; Fig 2C). Mice trained with stimuli 0.25 octave apart showed significant lower avoidance in nose-poking in conditioned visits (Multiple comparison on above ANOVA, p<0.05) and higher avoidance in the safe visits compared to mice trained with ΔF of 1 octave (Multiple comparison on above ANOVA, p <0.05). This deterioration in performance was more pronounced for the safe visits compared with conditioned visits (29.44% vs. 4.93% with respect to the equivalent safe and conditioned visits in the group trained with ΔF of 1 octave).

Notably, since rodent discrimination thresholds lie typically somewhere between 3% and 6% ΔF, all the tested stimulus-pairs were easily discriminable [11,12,22,24,26,27]. Yet, the frequency distance between the conditioned and the safe tone still affected discrimination performance dramatically. This wide-reaching effect of one stimulus over the other was possibly caused by the nature of the task, i.e. the fact that decisions were not based on the comparison between the present sound and an immediately preceding one but rather on the comparison between the present sound and the memory of previously presented sounds. These results suggest that the dynamics of learning in memory-based discriminations depend mainly on the physical distance between the trained stimuli.

### The psychometric threshold shifts towards the safe tone as ΔF decreases

The valence associated with a stimulus influences the width of generalization such that often negative stimuli generate more generalization around them than positive stimuli [28]. Here we quantified the effect of the ΔF between the safe and conditioned tones on the width of generalization. As before, once mice reached stable discrimination between the safe and conditioned tones, we tested their responses to novel tones. As was the case with the largest ΔF, during generalization testing mice maintained stable responses to the safe and the conditioned sounds (S3B, S3D and S3F Fig, open blue and red dots respectively). Animals avoided nose-poking during the first visits in which a novel sound was presented (S3A, S3C and S3E Fig, 1^st^ novel), and later responded to other novel sounds according to their frequency similarities to either the safe or conditioned sound (S3B, S3D and S3F Fig, testing). We found that shortening the frequency distance between trained stimuli narrowed the generalization around both safe and conditioned stimuli. This can be seen in Fig 3A and 3B where the flat portion of the psychometric curve becomes shorter as the ΔF becomes smaller. There was however a shift towards the left in the psychometric curve (Fig 3C) as demonstrated by a decrease in threshold (Fig 3D). Threshold differences were tested using a one-way ANOVA, which revealed an effect of training ΔF (*F*(3,63) = 9.9, *p* = 0.00002). Multiple comparisons revealed a significant difference between: ΔF 0.25 vs. 0.75 and 1; 0.5 vs. 0.75. The shift indicates that generalization was no longer symmetrical around the safe and conditioned tones for small ΔFs, but rather was relatively narrower around the safe tone. No change in slope was observed (Fig 3E). A one-way ANOVA on slope values revealed no effect of training ΔF (*F*(3,63) = 1.04, *p* = 0.383).

To quantify the relative change in generalization, we estimated the generalization width in octaves for individual mice, defined as frequency difference to the safe or conditioned stimuli eliciting more than 15% change in normalized performance (Fig 3F). Corroborating what we saw in the psychometric curves, for both safe and conditioned stimuli mice showed narrower generalization width (in octaves) as the safe and conditioned stimuli became considerably close, although it was comparable for safe-to-conditioned ΔFs above 0.5 octave. Smaller ΔFs, led also to significantly wider generalization around the conditioned stimuli than around the safe stimuli (Fig 3G). A 2-way ANOVA on generalization width values revealed an effect of training ΔF (*F*(146,3) = 90.25, *p* = 0), an effect of tone (safe or cond; *F*(146,1) = 0.23, *p* = 0.63), and an interaction (*F*(146,3) = 4.76, *p* = 0.0034).

Multiple comparisons yielded a significant difference between ΔF of 0.25 vs 0.75 and 1, 0.5 vs 0.75 and1, and 0.75 vs 1.

When generalization width was measured, not in octaves, but as a percentage of the safe-to-conditioned tone distance, the pattern changed (Fig 3H). The relative generalization width around the conditioned sound was, unlike the absolute generalization measured in octaves, constant across ΔFs with a value of about 30% (1-way ANOVA of relative generalization width on ΔF: *F*(3,73) = 1.04, *p* = 0.38). However, the relative generalization width around the safe sound was significantly lower for mice trained with ΔFs below 0.5 octave (1-way ANOVA of relative generalization width on delta f: *F*(3,73) = 12.79, *p* <0.0001. Multiple comparisons revealed a significant difference between ΔF 0.25 vs. 0.75 and 1; 0.5 vs. 0.75 and 1). Thus, a decrease in the safe-to-conditioned ΔF does not have an effect on the relative generalization around the conditioned sound, nor on the slope of the generalization but it has an effect on the level of generalization around the safe sound.

Generalization was not limited to frequencies in between the safe and conditioned tones, but also happened for flanking frequencies which were either below the safe tone or above the conditioned tone. Within the range of the frequencies tested, animals showed similar performance in response to the flanking frequencies close to the safe tone independently of which ΔF was used (S3B, S3D and S3F Fig). However, we observed, as others have done before [29,30], a shift in the peak of the generalization gradient for the conditioned tone for mice trained with a ΔF of less than 1 octave. As shown in S3 Fig, the avoidance response was strongest at the conditioned tone for mice trained with ΔF of 1 octave, but was displaced from the conditioned tone away from the safe tone for other groups. This is further indication that an interaction between the safe and the conditioned tone happened only for lower ΔFs.

Animals trained with lower ΔF performed a relatively larger number of short safe visits without nose-pokes (S4A Fig), paralleled by a larger number of daily visits in the conditioned phase (S4B Fig) and a concomitant larger frequency of visits (S4C Fig).

### Neuroligin 2 knockout mice showed impaired discrimination performance but normal generalization gradient

A smaller safe-to-conditioned ΔF results in an increase in the number of safe visits without nose-pokes (baseline response) and a narrower generalization around the safe sound. The upward shift in the baseline responses could, alone, be a byproduct of increased anxiety associated with a more difficult task, i.e. stressed mice tend to be more cautious [31,32]. We predicted that, if this is the case, increasing anxiety without decreasing the safe-to-conditioned ΔF would result in an increased baseline. Therefore, we tested generalization in Neuroligin 2 knockout (KO) mice, as a model of anxiety. Neuroligin 2 (Nlgn2) is a synaptic adhesion protein that is thought to be exclusively localized at inhibitory synapses [33]. The loss of function of Nlgn2 in mice leads to relative selective increase in behavioral anxiety [21,34]. In this experiment, the safe sound was 7000 Hz and the conditioned, 14000 Hz. Nlgn2 knockout mice showed comparable baseline behavior compared to WT littermates (Fig 4A and 4B, baseline), and normal distribution of visit lengths for safe and conditioned visits with and without nose-pokes (S7A–S7B Fig). Unexpectedly though, when conditioning began, Nlgn2 knockout mice did not show an increased baseline but, instead, showed less avoidance during conditioning visits than WT animals (Fig 4A and 4B, red line). Interestingly, the effect that the first two dozen conditioned visits had on subsequent safe visits, reflected in the increase in avoidance during safe visits following conditioned visits (see blue line in Fig 4A and, as previously described, Fig 2A), was absent in the Nlgn2 knockout mice. The overall effect was a reduction in d’ in these mice, which was below 1 throughout the time of testing (Fig 4C; 2-way ANOVA of d’ on training days and genotype revealed main effects on training days, *F*(7,135) = 3.61, *p* = 0.0014; and main effects on genotype, *F*(1, 135) = 101.96, *p* = 0). But while the decrease in d’ in animals trained with small ΔFs was a result of an increase in avoidance during safe visits, in the Nlgn2 knockout mice it was caused by a decrease in avoidance during conditioned visits (92.98% avoidance for the WT, 72.26% for Nlgn2 KO; unpaired t-test for the mean performance during the last 3 days of conditioning, *p* < 0.0001).

This was not the result of increased generalization around the safe tone, since the shape of the psychometric curves in Nlgn2 knockout and WT mice was very similar despite the very different end points (Fig 4D; full two-way ANOVA model revealed a significant effect of genotype (Nlgn2 knockout vs WT, *p* = 0.0005), significant effect of stimuli (*F*(9,140) = 79.61, *p* = 0), and no interaction (*p* = 0.17)). There was no significant difference in the psychometric threshold between the groups, which was equidistant from the safe and conditioned tones (Fig 4E and 4F; unpaired t-test, *p* = 0.21; average threshold for Nlgn2 knockout is -0.048; for WT is 0.018). This indicates that, like in the WT, the Nlgn2 knockout mice generalized equally around the safe and conditioned tone and that the width of the generalization was similar to that of WT animals. There was also no difference in the slope of the psychometric curve between the groups (Fig 4G; *p* = 0.56).

Thus, Nlgn2 knockout mice showed impaired discrimination during learning and yet both the slope and width of the generalization gradient were comparable to that of WTs, indicating that while valence assignment in these mice is affected, sensory acuity is not. These results indicate that a genotype associated with increased anxiety does not necessarily lead to an increased avoidance in safe visits, nor to asymmetrical generalization width around the safe and conditioned tones.

### The direction of conditioning along the frequency axis influences discrimination learning but not generalization

Neurons in the auditory system often have asymmetrical tuning curves, with shallower slopes for frequencies below that which elicits the strongest response [35]. Potentially, this asymmetrical tuning could have an effect on discrimination learning and generalization [36]. Since the conditioned tone was above the safe tone in the experiments described so far, the shallower tuning towards the safe tone might have affected the pattern of results. We now trained three groups of mice with ΔF of either 0.75 or 1 octave in the Audiobox as we did before but using the low frequency tone as conditioned and measured avoidance during conditioned visits as well as during safe visits before and after conditioning began (Fig 5A–5C; right data are replotted from the subset in Fig 2C which was trained with corresponding frequencies). Overall mice discriminated better when the conditioned tone was higher in frequency than the safe tone. Combining all groups as depicted in Fig 5A–5C, a 3-way ANOVA on the number of visits without nose-pokes revealed a significant main effect of safe vs conditioned visits (*F*(1,100) = 765.46, *p* = 0), a main effect of whether conditioned tone was higher or lower than the safe one (*F*(1,100) = 11.96, *p* = 0.0008), and a significant interaction between the two (*F*(1,100) = 10.93, *p* = 0.0013) but no effect of tone-pair (*F*(1,100) = 1.76, *p* = 0.19). When the comparison was made on the d’ values (Fig 5D), a 2-way ANOVA revealed an effect of the relative position of the conditioned tone (*F*(42,1) = 12.5, *p* = 0.001), but no effect of tone-pair (*F*(42,2) = 0.75, *p* = 0.48) or interaction (*F*(42,2) = 0.89, *p* = 0.42). Individual 2-sample t-tests yielded a significant difference in the relative position of the conditioned tone only for the tone pair 6–12 kHz (*p* = 0.004; Fig 5D, middle).

Generalization on the other hand was overall more invariant (Fig 5E–5G). Only in one of the three groups, trained with 1 octave ΔF (safe = 14000 Hz and conditioned = 7000 Hz), did we find a significant shift in the psychometric threshold towards the conditioned sound compared to the opposite group (Fig 5E).

These findings suggest that discrimination learning is influenced by both the ΔF and the polarization, whereas for generalization only the first one matters. These data support again the idea that generalization gradients depend strongly on the physical distance of the trained stimuli.

### Previous experience with the task did not facilitate discrimination but shifted generalization gradients

We investigated how previous experience in the Audiobox influenced learning of new conditioned sounds, existing generalization gradients, and discrimination acuity. After training and testing animals with one pair of safe and conditioned tones, we replaced the initial conditioned tone for a novel conditioned tone, without changing the safe tone. Mice were then trained with only the safe and new conditioned tone for a few days before generalization curves with intermediate tones were measured. In this second phase of generalization testing, the first conditioned tone was not conditioned anymore (Fig 6A).

First, we compared the performance of mice that had been trained with a ΔF of 1 and 0.5 octaves before being retrained with, respectively, a ΔF of either 0.5 or 1 octave (Fig 6B and 6C, right), with the performance of naïve mice trained directly with a ΔF of either 0.5 or 1 octave (Fig 6B and 6C, left). Mice in all groups showed similar responses to the safe tone (unpaired t-test, *p* > 0.05 for all groups). Interestingly, despite their different training history, mice first conditioned with an easier high ΔF (1 octave; Fig 6B right) did not show better discrimination relative to naïve animals when further conditioned with a lower ΔF (0.5 octave; Fig 6B). This suggests that there is no knowledge transfer from an easy to a hard discrimination. When we first trained mice with a more difficult low ΔF (0.5 octave), however, there was also no facilitation of a subsequent discrimination with a high ΔF (1 octave, Fig 6C right) relative to naïve mice (Fig 6C left). Performance was, in fact, subtly but significantly worsen (unpaired t-test, *p* = 0.016). Overall the data suggest that previous training did not facilitate subsequent discrimination learning.

We then investigated how generalization was shaped by previous experience. We compared the generalization gradients of naïve and experienced mice and measured the psychometric threshold (Fig 6D–6F). The tone that was first conditioned is marked in the generalization gradient but is no longer conditioned during this second generalization testing. In Fig 6D and 6E it is evident that previous training has an effect on the generalization gradient, which is subtly different from that in naïve mice. Indeed, the psychometric threshold, measured here in frequency values rather than relative distance as before, was significantly different from that of naïve mice (Fig 6F; unpaired t-test, conditioned at 9.8Hz, *p* = 0.006; conditioned at 14kHz, *p* = 0.0002). There was no significant difference in slope (Fig 6G). Interestingly, the order of conditioning did not influence the final slope and width of generalization (Fig 6H). Animals conditioned with a 14 kHz tone followed by 9.8 kHz showed similar psychometric threshold than animals trained in the reversed order, first 9.8 kHz then 14 kHz (unpaired t-test, *p* = 0.47). This is because although previous experience did not lead to better discrimination, conditioning a lower frequency than that used during training (9.8 kHz after 14 kHz; Fig 6E) elicited a shift in the psychometric curve compared to that generated during the initial training. Generalization is a summation of the animals training history.

We then compared the first and second generalization gradient for the same animals after the first and second conditioning respectively. More groups were included in this analysis, with ΔF ranging from 0.125 to 1 octave. In all groups, we found that retraining shifted the psychometric curve towards the second conditioned frequency (S6A–S6E Fig, leftmost column). This effect was especially strong in animals that were initially trained with ΔF above half an octave and retrained to a lower ΔF (S6A, S6C and S6D Fig, middle column; comparing thresholds; paired t-test, *p*_*s7A*_ = 0.007, *p*_*s7C*_ = 0.0005, *p*_*s7D*_ = 0.005). For mice first trained with relatively small ΔF 0.25 octave and retrained to a smaller ΔF, 0.125 octave, their psychometric thresholds tended to shift but without statistical significance (S6E Fig, middle column; paired t-test, *p* = 0.15). Conditioning the mice to a higher ΔF than the first conditioning also moved the psychometric threshold away from the safe tone but with no statistical significance (S6B Fig, middle column; paired t-test, *p* = 0.053). For all groups, we found that there was no consistent change in slope (S6 Fig, rightmost column; paired t-test, *p*_*A*_ = 0.57, *p*_*B*_ = 0.16, *p*_*C*_ = 0.16, *p*_*D*_ = 0.81, *p*_*E*_ = 0.33). These results confirmed that generalization gradients for animals trained with multiple conditioned sounds reflects the history of training and not simply the level of avoidance of the last conditioned tone.

### Discrimination acuity was increased around the conditioned tone after Audiobox learning in a ΔF-specific manner

In order to test whether discrimination training in the Audiobox led to changes in sensory perception we tested acuity around both the conditioned and safe tones. To avoid task-specific influences we tested acuity using the pre-pulse inhibition (PPI) of the acoustic startle response (ASR) protocol. We wanted to understand, for example, whether the narrowing in the generalization gradient observed in animals trained with a small ΔF was a result of a change in discrimination acuity per se. In the PPI protocol, ASR can be partially inhibited by a preceding warning pre-pulse tone. The inhibition effect of the pre-pulse tone on ASR magnitude is highly depended on the saliency of this tone. If a constant background tone is present, the disparity in frequency between the background and the pre-pulse tone will determine the efficacy of the latter in inhibiting the ASR (see Methods, S7A and S7B Fig; [22,23,37]). Discrimination acuity was quantified in terms of frequency discrimination threshold, defined as the difference between background and pre-pulse frequency that generated 50% of the maximum inhibition (see Methods, S7C Fig). Since there are innate differences in discrimination acuity for different frequencies, we focused on the change in discrimination thresholds triggered by training (before versus after) and tested individual animals using either the Audiobox safe or conditioned tones as the background (f1, constant across the two PPI tests for a given animal). We focused on groups with safe-to-conditioned ΔF of 0.25 and 1 octave, which were trained with 7 kHz as safe and either 8.32 kHz or 14 kHz as conditioned.

Training led to different effects on discrimination acuity around the safe and conditioned sounds. Animals in both groups, showed a significant increase in inhibition that was surprisingly specific for the tone 0.03 octave above the safe tone (Fig 7A and 7B, top). The overall discrimination threshold around the safe tone tended to decrease, but without statistical significance (Fig 7C and 7D, 7-up; paired t-test, *p* = 0.17). Thus, training tends to improve discrimination acuity around the safe tone, but only for frequencies above this tone (towards the conditioning tone). Animals in the 0.25 ΔF group showed a non-significant tendency for decreased PPI for pre-pulse tones around the 8.32 kHz conditioned tone (Fig 7A, bottom) and increased threshold (Fig 7C, 8-down; paired t-test, *p* = 0.10). The strongest effect was seen for pre-pulse tones when the 14 kHz conditioned tone was used as background in the 1 octave group. Following Audiobox discrimination training PPI was significantly enhanced for pre-pulse tones both above and below the 14 kHz conditioned tone (Fig 7B, bottom). The result was a significant decrease in threshold around 14 kHz that was more pronounced for pre-pulse tones below the conditioned tone (towards the safe tone; Fig 7D, 14-down; paired t-test, *p* = 0.03).

We then investigated how subsequent conditioning affected the change in acuity induced by the initial conditioning (Fig 7E, top). For mice that were initially conditioned with a 14 kHz tone and subsequently conditioned with a 9.8 kHz tone, we found that the effect of initial conditioning on acuity was partially reversed by the second conditioning (Fig 7E and 7F). This suggests that the modulation of acuity induced by learning is very dynamic.

Taken together, wider generalization gradients in the Audiobox were not the result of diminished perceptual discrimination. On the contrary and paradoxically, in the high ΔF group, the wider generalization observed in the Audiobox was accompanied by increased discrimination acuity. In turn, narrower generalization in the low ΔF group was not accompanied by improved acuity.

## Discussion

In the present study, we assessed learning and generalization in C57BL/6J mice in a tone frequency discrimination task in an automatic and naturalistic environment, the Audiobox. The task required the mice to make memory-based decisions and had the characteristics of a go/no-go discrimination task. We investigated how (1) the frequency distance between trained stimuli, and (2) the mice past experience with these stimuli, affected learning speed, discrimination performance and generalization gradients. First, we found that the physical distance between stimuli, the difference in frequency between the safe and conditioned sounds, was the main contributor to discrimination performance while the past experience with the same sounds exerted a weaker influence. Second, we found that while the generalization slope was constant throughout the different manipulations, generalization width, was influenced both by the physical distance between the to-be-discriminated tones and the mice past experience. Third, valence had a modulatory influence on the generalization width only when the distance between the safe and conditioned sounds was decreased such that task difficulty was increased. In this case, although generalization was overall narrower, it became asymmetrically wider around the conditioned sound. Fourth, generalization around a sound did not reflect a decrease in sensory acuity around the same sound.

In conclusion, the tonotopic organization of the auditory system in mice is the main determinant of discriminative task performance in the auditory processing. In relative judgement tasks, where discrimination is based on the difference between the current stimulus and the one immediately preceding, the history of activation of the involved neuronal populations is likely to have a strong impact on discrimination. In fact, pure tone discriminations are typically easy for mice and do not require the auditory cortex [38,39]. Memory-based discriminations might be inherently harder [19] because the pattern of activity triggered by, say, the conditioned tone cannot be directly compared to the pattern of activity triggered by the presentation of the safe tone. Based on our results, we conclude that performance in memory-based discriminations relies on wide sensory filters. By this we mean that current performance is influenced by previously experienced stimuli even when these differ substantially, in frequency in our case, from the current one. One possible explanation is a strong influence of the wide tuning typical of subcortical structures [35,40].

In our study, we reliably measured leaning speed, discrimination performance and generalization gradients through an Audiobox paradigm that mimics the natural environment to the extent that it is possible in a small-scale well-controlled experimental setting. As mentioned in a previous study [24], the Audiobox allows training and testing in the animal’s living quarters. Mice live for days at a time in groups of 8 to 10 subjects and are neither food- nor water- deprived, mirroring the social environment that is natural for rodents. Moreover, the automatic detection system (see methods) allows mice to initiate stimulus exposure at will, unlike in other operant protocols [1,41]. Task attendance was driven by innate curiosity and water demand, allowing us to simplify the procedural learning. Overall, this led to fast learning and stable performance.

The animal’s innate curiosity results in a high number of corner visits per day (on average 110 during conditioning and 150 during generalization phases). As a result, there is a high false alarm rate: in about one third of safe visits mice do not nose-poke. Performance was quantified using nose-poking behavior, but visit duration correlates well with nose-poking, with the shortest visits (about 3 seconds) being those that are accompanied by the conditioned sound.

The present data show that learning speed, discrimination performance, and generalization gradients are dependent on the physical distance between the trained stimuli. Smaller safe-to-conditioned ΔF elicited slower learning, worse discrimination and narrower generalization gradients around trained stimuli. Just-noticeable differences in mice were previously reported to be around 2–5% in relative judgement tasks [11,22,26] or 4–7% in a comparable memory-based task [24], all well below the ΔFs used here. Had the main constrain in our task been the discriminability of the sounds, we would have expected discrimination performance to remain at high-level and only drop, relatively suddenly, when the ΔF was near the JND. What we found, however, is that even as the ΔF decreased from 1 octave (100%) to 0.25 octave (19%), both well above the JND, discrimination performance deteriorated dramatically. This could mean that the internal representation along the frequency axis of the safe and conditioned sounds, which is probably the basis of memory-based discriminations, interact with each other in ways that influence this type of task performance. The increase in avoidance during safe visits under small ΔF conditions could be caused by the internal representation of the conditioned tone encompassing to a certain extent that of the safe tone. Since the effect is already observed in the group trained with the 0.75 octave ΔF, the width of this internal representation extends at least 0.75 octave below the conditioned sound. This is unusually wide given the critical bandwidth in the auditory cortex of C57BL/6 mouse [42,43] but is comparable to the generalization width of latent-inhibition observed using the same Audiobox paradigm in a previous study [24].

Mice showed better discrimination performance when the conditioned tone was of higher frequency than the safe tone. This asymmetry in behavior is unlikely the result of a differential innate/prior knowledge about the stimuli’s frequency, since baseline activity was comparable across all tested frequencies. We hypothesize that the asymmetry was caused by an asymmetrical stimulus generalization, such that the generalization around the conditioned tone was stronger towards the higher frequency. Similar asymmetrical generalization has been reported by Bang et al. [36] in rat using fear conditioning. They found that rats showed differential responses to the CS+ and CS- only when 19 kHz pips, but not 4 kHz, were conditioned, although both sound stimuli were equally neutral before conditioning and elicited similar level of freezing behavior.

Overall the data support the hypothesis that perception of isolated stimuli (in a memory-based task) is constrained by a pre-wired circuitry underlying auditory processing. The uncommon wide and asymmetrical generalization, in line with our previous results [24] and those of others [43,44], suggests that pure tone frequency perception may be determined by the tonotopic organization of peripheral and subcortical areas, where neurons with wide and asymmetrical tuning curves have been recorded [35,40]. The fine acuity of behavioral discrimination reflected in low JNDs values can be achieved through the integration of information across wide tuning curves, as has been suggested for both the visual and the auditory system [15,45–47]. The steep slopes of tuning curves can also convey substantial information about frequency differences [40,48]. That these comparisons modulate behavior is reflected in the effect that previous history has on current performance [26,49]. In either case, the extraction of discriminative information from curve integration or tuning slopes relies on the comparison across responses elicited by different frequencies, something that is not possible in the same manner in a memory-based task in which the response to at least one of the frequencies needs to be pulled from memory. Neurons with ultra-fine tuning have been described in the auditory cortex of humans [50]. While these are not the norm, it is also not clear how their activity would help since a memory trace of previous activation would rarely overlap with the current response.

A stimulus of negative valence tends to have stronger behavioral and psychological impact on aspects such as speed of task learning [51], generalization width [28,52] and perceptual learning [37,53–56]. As a result, we would have expected wider generalization gradient towards the conditioned stimuli than the safe stimuli. Our findings are consistent with this view but only for ΔF below 0.75 octave, suggesting that valence has a secondary modulation influence on generalization that becomes visible only as the discrimination becomes more difficult.

Responses towards the safe and conditioned tones varied differently with changes in ΔF. While for the conditioned tone decreases in ΔF affected mainly the speed of avoidance learning but not the final level of avoidance, for the safe tone the same changes had a dramatic effect on the final level of nose-poking response. Also, smaller ΔF had a more prominent narrowing effect on generalization gradients around the safe tone. Thus, positive and negative associations affect behavior differently. Physiological data suggest that negative and positive associations are processed in distinct but overlapping networks [57–59]. In mice, recent research on the sense of taste found that anatomically separated projections imposed different valence on sweet or bitter tastes [60].

Anxiety influences learning and generalization [32,52,61]. In the PPI data, acoustic startle responses were larger in mice trained in the Audiobox with a smaller ΔF. Since greater startle reflex is associated with higher level of anxiety [62], the increased startle with smaller ΔF could reflect the differential level of stress in the Audiobox between the groups trained with small ΔF and those trained with larger ΔF. This might have caused the shift in the generalization gradient. However, no shift was observed in the Nlgn2 knockout mice, a model of anxiety [21,63]. These mice showed decreased avoidance of the conditioned tone, a finding consistent with recent observations of learning impairments in mice that have an equivalent knockout of the Nlgn2 protein [64,65]. However, and independent of possible learning impairments, mice lacking Nlg2 are highly anxious, indicating that overgeneralization does not always accompany increased anxiety. The asymmetrical generalization of animals trained with small ΔFs need not be the result of increased anxiety levels, a finding inconsistent with studies that have suggested that overgeneralization can underlie anxiety disorders [31,66].

In our paradigm, mice did not benefit from their past training experience when a new conditioned tone was introduced, independently of whether this increased or decreased the new safe-to-conditioned ΔF distance. After re-training mice reached similar, sometimes even worse, performance than mice without prior training (e.g. animals trained first with 14 kHz as conditioned tone and then with 9.8 kHz, versus mice trained directly with 9.8 kHz as conditioned tone). Human and animal studies on learning transfer under different stimulus classes suggest that the influence of initial training is based on implicitly learned integration of information about the stimuli, procedure, and cognitive skills [1,67–69]. In our task, the different aspects of the experimental design (group housing for long period of time, *ad libitum* performance, absence of deprivation and automatic detection) meant that the task required very little procedural learning on the part of the mice. This may explain the small effect from past experience on subsequent training.

Generalization, however, was found to be affected by the summation of the animals’ training history, consistent with findings obtained in pigeons on light wavelength generalization [70]. However, it is interesting that this summation effect did not depend on the chronological order of training, since we observed identical generalization gradients in mice trained with 9.8 and 14 kHz, independently of which tone was conditioned first.

Training in the Audiobox had an effect on discrimination acuity measured in a subsequent PPI test. In line with previous studies [55,71,72], we saw a moderate improvement in acuity around the safe tone. Aversive learning, however, only led to an improvement in acuity around the conditioned tone when the safe-to-conditioned ΔF was large. Overall, the wider generalization of the large ΔF, but not the narrow generalization of small ΔF, was accompanied by increased acuity. Our results are not consistent with the view that over-generalization results from a decrease in discrimination acuity caused by aversive learning [28,55,73]. For example, in a recent study in which discrimination acuity was measured in the same way, wider generalization was accompanied by decreased acuity, whereas a training protocol that induced narrow generalization led to increased acuity following aversive learning [37]. It is possible that the decreased acuity observed in other studies following aversive learning is a result of other factors, such as the use of different training paradigm (fear conditioning vs. avoidance learning) that might invoke different brain mechanisms. Fear conditioning may rely on a fast, pre-attentive, amygdala-based, system [74–76], whereas avoidance learning may rely on a more complex, cortical system [77]. Indeed, the physiological changes induced by classical or instrumental conditioning vary, which leads to the argument that plasticity also depends critically on the task characteristics [78].

The modulation in acuity is probably local and dynamic as suggested by the fact that retraining to a new conditioned tone led to a reversal of the observed changed in acuity. Physiological plastic changes associated with behavioral learning have also been suggested to be very dynamic [79]. The change in discrimination acuity might be epiphenomenal and not serve as the substrate of improved performance or learning itself [80–82].

In conclusion, decisions based on memory, i.e. in the absence of a recently perceived comparative stimulus, are constrained by the physical distance between the to-be-discriminated stimuli. This suggests that wider representation filters are in place during memory-based decisions. As the ΔF between the trained stimuli diminishes, safe and conditioned tones activate progressively more overlapping neuronal populations. In a relative judgement task, the recent activation of one neuronal population (responding to a safe sound, for example) might help detect the differences in firing pattern of the currently activated neuronal population (responding to a conditioned sound, for example). Mechanisms such as adaptation of the recently activated neurons would decrease the overlap in the firing of the two populations and enhance the difference. In a memory-based task, however, the discrimination must rely on the memory of the representation of the safe and conditioned sounds. Based on our data, we argue that the memory traces for ΔF of 0.5 octave or less overlap sufficiently for discrimination to become difficult. In this situation, the animal becomes more conservative in its decision and its behavioral responses are more cautious. That discrimination is substantially impaired already at ΔF of 0.5 octaves suggest a strong role of pre-wired tonotopic organization and the involvement of subcortical areas, with wider tuning curves [35]. Nevertheless, generalization of training to other similar stimuli depends on an interaction between the conditions of the training (ΔF), stimulus valence and past experience. This suggests that to perceptually distinguish stimuli (discrimination) and to respond to similar stimuli based on prior knowledge (generalization) are two processes that may rely on somewhat distinct circuits.

## Supporting information

S1 File Minimal data set for Figs 1 to 7. Each sheet contains the minimal data used for each plot.
(XLSX)

S2 File Minimal data set for S1 to S7 Figs. Each sheet contains the minimal data used for each plot.
(XLSX)

S1 Fig Memory-based discrimination protocol and Audiobox apparatus. (**A**) Photos (left) and schematic representation (Right) of the Audiobox. (**B**) Shema of a single safe/novel (top) and conditioning (bottom) visit. Subjects initiated a visit by entering into the corner. Pure tone pips of fixed frequency presented for the duration of each visit predicted whether nose-poking was followed by access to water (top) or an air-puff (bottom). **(C)** Number of visits per day per animal during the safe phase. **(D)** Cumulative distribution of inter-visit-intervals in hours during the safe phase. Dash line is on the 1 minute mark. **(E)** Distribution of visit lengths during the conditioned phase for safe (left) and conditioned (right) visits with (black) and without (white) nose-pokes. Mean overall length is in writing.
(EPS)

S2 Fig Individual performance and visit length analysis. (**A**) Average response to the safe (blue), conditioned (red) and novel (black) tones as a function of tone frequency for individual mouse. Bottom right: mean responses. (**B**) Mean visit length of the safe (blue), conditioned (red) and novel (black) visits as a function of tone frequency. Calculation was done separately for visits with (closed dots) and without (open dots) nose-pokes. (**C**) Normalized inversed visit length (black dots) and fitted psychometric curve (red). Dash line: psychometric threshold or stimulus strength for which performance is at the midpoint. **(D)** Cumulative distribution of short (up to 60 seconds) inter-visit-intervals in seconds during the conditioned phase. Black vertical line is on the 10 second mark.
(EPS)

S3 Fig Generalization test for mice trained with different ΔF. (**A**) Mean daily performance for the safe (blue, 7000 Hz), conditioned (orange, 11770 Hz), and novel (color based on the frequency) visits during generalization test. Group trained with ΔF of 0.75 octaves. (**B**) Average performance across all visits as a function of tone frequency. Responses to the safe (blue open circles), conditioned (red open circles) tones averaging over testing days of each novel visit type. The triangles on the x-axis indicate the frequency of the safe (blue) and conditioned (red) tone. (**C-D**) Same as (**A-B**) for mice trained with 7000 Hz safe tone and 9800 Hz conditioned tone. Group trained with ΔF of 0.50 octaves. (**E-F**) Same as (**A-B**) for mice trained with 7000 Hz safe tone and 8320 Hz conditioned tone. Group trained with ΔF of 0.25 octaves.
(EPS)

S4 Fig Visit characteristics for different training ΔFs. **(A)** Distribution of visit lengths during the conditioned phase for safe (left) and conditioned (right) visits with (black) and without (white) nose-pokes. Mean overall length is in writing. **(B)** Mean number of visits per day per animal during the safe, conditioned, and testing phase for the different groups, color-coded by training ΔF. **(C)** Cumulative distribution of inter-visit-intervals in hours during the safe, conditioned and testing phases for the different groups, color-coded by training ΔF. Dash line is on the 1 minute mark.
(EPS)

S5 Fig Visit length in Nlgn2 mice. Distribution of visit lengths for **(A)** Nlgn2 wild type mice and **(B)** Nlgn2 KO during the conditioned phase for safe (left) and conditioned (right) visits with (black) and without (white) nose-pokes. Mean overall length is in writing.
(EPS)

S6 Fig Retraining to another conditioned tone shifted psychometric threshold. (**A-left**) Average generalization gradients following the first (purple; ΔF 1octave) and the second (gray; ΔF 0.5 octave) conditioning. (**A-middle and right**) Threshold and slope calculated from fitted the psychometric curve. (**B-E**) Same as (**A**) for the remaining groups. The safe (open circle) and conditioned (closed circle) tone used in each conditioning was marked respectively. The gray arrow indicates the direction in which the second conditioned tone moved away from the first one. The safe-to-conditioned ΔF for each generalization gradient was shown in the label.
(EPS)

S7 Fig Acoustic startle apparatus for frequency discrimination acuity measurement. (**A**) Scheme of the acoustic startle setup (top) and a single PPI trial (bottom). PPI protocol consisted of three stimuli: background tone (f1), pre-pulse tone (f2) and startle noise that evoked a startle response. On each trial, a pre-pulse tone with a frequency shift of between -0.56 and 0.4 octave from the background tone was pseudo-randomly chosen from 13 frequencies. (**B**) Example average traces for one mouse represented the force measured on the platform during the PPI test for each pre-pulse tone. Background tone (labeled in red, f1) was 14000 Hz. The magnitude of the startle response decreased as the frequency shift between the background and pre-pulse tone became bigger. (**C**) Sample PPI curve for naïve mice (n = 10) tested with background tone of 14000 Hz. Red line is the logistic fit curve (see Methods). Discrimination threshold (-0.173 and 0.022 octave for frequency below and above f1, respectively) was defined as a frequency shift that elicited 50% of the maximum inhibition (dash line).
(EPS)
